# Using discrete choice experiments as a decision aid in total knee arthroplasty: study protocol for a randomised controlled trial

**DOI:** 10.1186/s13063-016-1536-5

**Published:** 2016-08-19

**Authors:** Michelle M. Dowsey, Anthony Scott, Elizabeth A. Nelson, Jinhu Li, Vijaya Sundararajan, Mandana Nikpour, Peter F. M. Choong

**Affiliations:** 1 Department of Surgery, The University of Melbourne, St. Vincent’s Hospital, Level 2 Clinical Sciences Building, 29 Regent Street, Fitzroy, Melbourne, 3065 VIC Australia; 2 Melbourne Institute of Applied Economic and Social Research, The University of Melbourne, Melbourne, Australia; 3 Department of Orthopaedics, St Vincent’s Hospital, Melbourne, Australia; 4 Department of Medicine, St Vincent’s Hospital, The University of Melbourne, Melbourne, Australia; 5 Departments of Medicine and Rheumatology, St. Vincent’s Hospital, The University of Melbourne, Melbourne, Australia

**Keywords:** Total knee arthroplasty, Discrete choice experiment, Risk perception, Patient expectations, Outcomes

## Abstract

**Background:**

Osteoarthritis (OA) is a leading cause of disability in developed nations. Total knee arthroplasty (TKA) is a clinically effective treatment for people with end-stage knee OA, and represents one of the highest volume medical interventions globally. However, up to one in three patients remain dissatisfied following TKA. Research indicates that the strongest predictor of patient dissatisfaction following TKA is unmet expectations. This study will use a discrete choice experiment (DCE) provided to patients to improve knowledge of the expected outcomes of TKA. This increased knowledge is based on actual outcome data and is hypothesised to optimise patient expectations of TKA outcomes, thereby increasing their satisfaction and self-reported health outcomes.

**Methods/design:**

One hundred and thirty-two people with end-stage OA on the waiting list for TKA will be recruited and randomly allocated to one of two groups using computer-generated block randomisation. A randomised controlled trial (RCT) adhering to SPIRIT and CONSORT guidelines will evaluate the effect of administering a DCE prior to surgery on patient-reported pain and function and satisfaction following TKA. Patients in the intervention arm will complete a survey containing the DCE, compared to the control group who will complete a modified survey that does not contain the DCE activity. The DCE contains information on actual risks of postoperative complications, as well as health status after TKA. The DCE encourages patients to actively make trade-offs between risks and health outcomes to elicit their preferences. Participants in both groups will be required to complete the survey after consenting to have the procedure, but prior to surgery during their routine preadmission appointment at St. Vincent’s Hospital, Melbourne, Australia (SVHM). Patients in both the intervention and control groups will also be required to complete a brief patient expectation survey 1 week prior to scheduled TKA. In addition, orthopaedic surgeons will complete a brief expectations survey for each patient consented for TKA to compare matched surgeon and patient expectations for recovery following TKA. Primary outcomes will be evaluated by a blinded examiner at 12 months post surgery using a validated self-reported pain and physical function scale, and a validated patient satisfaction scale. Secondary outcomes will include a range of validated measures of health and psychological wellbeing. All analyses will be conducted on an intention-to-treat basis using linear regression models.

**Discussion:**

This study is the first of its kind to use a DCE to provide information to patients to optimise their expectations of the outcomes of surgery. Reducing the rate of patient dissatisfaction commonly seen in patients following TKA will help to reduce the burden associated with poor outcomes on the health system.

**Trial registration:**

Australian New Zealand Clinical Trials Registry (ACTRN12615001226594p). Version 1; registered on 9 November 2015.

**Electronic supplementary material:**

The online version of this article (doi:10.1186/s13063-016-1536-5) contains supplementary material, which is available to authorized users.

## Background

Osteoarthritis (OA) is one of the most disabling diseases in developed countries and is responsible for significant functional limitation in over 43 million people worldwide, 27 million of whom are 60 years of age or older [1]. Age is the strongest predictor of the development and progression of OA, and as such the number of people suffering with OA is expected to increase over the coming years due to population ageing [2]. Total knee arthroplasty (TKA) is a cost-effective treatment for people with end-stage knee OA [3] that improves quality of life by reducing pain, joint deformity and loss of function. It is a high-cost [4, 5] and high-volume procedure [6], which dominates national surgical waiting lists. The number of TKAs being performed each year has risen markedly over the past decade and on average has doubled in most Organization for Economic Co-operation and Development (OECD) countries [2]. In Australia, nearly 50,000 people underwent TKA in 2014 at an estimated cost exceeding Australian $1 billion [5, 6].

Many studies have confirmed the beneficial impact of TKA on pain, disability and quality of life on average [7, 8]; however, surgery is not without risk, and not without heterogeneity in outcomes. In the short-term postoperative period, there is a small risk of severe complications [9–11] and in the longer term there is the risk of prosthesis failure, primarily through loosening, resulting in the need for complex revision surgery [6]. While survival analysis is crucial for understanding the failure rate of TKA, revision surgery alone as a sole index of failure has been called into question because of the potential for underestimating the problem [12]. In this regard patients with joint pain and dysfunction may endure years of dissatisfaction without undergoing revision surgery, with reports suggesting that 15–30 % of patients are dissatisfied despite the procedures being technically and radiologically satisfactory [13].

Not all patients undergoing TKA are at equal risk of complications. A number of patient factors can increase the risk of complications for individuals undergoing TKA, including: age and gender [14], body mass index [9, 10], ethnicity [15], psychological distress [16, 17], baseline pain and functional disability [14], comorbidity profile [9, 14] and radiographic OA severity [14]. For individuals considering TKA as a treatment option very little is known about patients’ perspectives on acceptable level of risk and how closely aligned or not this risk is with their treating clinicians’.

Although patients and clinicians share similar goals of maximising treatment benefits while minimising risk, they may have different perspectives on trade-offs among benefits and risks of treatment [18]. The acceptable level of risk tolerated by a patient depends not only on the benefits provided by the TKA (significant pain relief and improvement in joint function) but also on the seriousness and severity of the disease, the availability of other treatments and other factors such as risk of complication [19]. To date, patients’ assessments of risks and benefits of surgical interventions have not been subjected to rigourous evaluation. This is important because unrealistic patient expectations and uninformed perceptions of potential benefits, risks and limitations of surgery lead to dissatisfaction following TKA [20].

It has been shown that more than one half of patients undergoing TKA have expectations of surgery that exceed those of their surgeons; in contrast, only one quarter expect less than their surgeons [21]. Patients with higher levels of expectations than their surgeons anticipated greater improvements in activity levels and those with lower expectations anticipated a higher incidence of complications. It has also been reported that the rate of dissatisfaction amongst TKA recipients with unmet expectations is as high as 49 % compared to 6 % in those whose expectations have been met [13].

This study will use a discrete choice experiment (DCE) as an intervention to provide patients with more realistic expectations of the outcomes of TKA, specified in terms of operative and postoperative risks and improved function and quality of life. DCEs are choice models that describe, explain, and predict choices between two or more discrete alternatives, such as whether or not to undergo surgery. The DCE is administered as part of a survey, where patients are asked to compare a series of hypothetical (but realistic) scenarios that describe risks and outcomes of TKA, and asked to choose which they would prefer [22]. By asking individuals to choose between scenarios, the DCE elicits patients’ willingness to accept trade-offs between features of specific treatments with different characteristics, thereby revealing their preferences in terms of the relative importance of each characteristic of the treatment [23, 24]. The impact of administering a DCE prior to TKA on quality of life and satisfaction post surgery will be examined in a randomised controlled trial (RCT).

To date, patients’ assessments of risks and benefits of surgical interventions, and specifically TKA, have not been subjected to rigourous evaluation. The aim of this research is to use DCEs to (1) explore how patients with end-stage OA undergoing TKA balance risks and benefits, (2) evaluate the effect of information from the DCE on health outcomes and satisfaction following TKA, and (3) compare matched surgeon and patient expectations for recovery following TKA.

## Methods

### Experimental design

The design is a two-arm, blinded, randomised controlled clinical trial comparing satisfaction and health outcomes of TKA patients who are provided with a DCE prior to undergoing TKA, with that of a control group. Patients in the intervention arm will complete a survey containing the DCE. Patients in the control arm will complete a survey that does not contain the DCE activity. Patients will also complete a brief patient expectation survey 1 week prior to scheduled TKA. Outcomes will be compared at 12 months after TKA. In addition, orthopaedic surgeons will complete a brief expectations survey for each patient consented for TKA. This will allow for a matched surgeon/patient comparison of expectations for recovery following TKA. The study strategy is registered, constructed and presented according to the recommendations of the Standard Protocol Items: Recommendations for Interventional Trials (SPIRIT) [25] (see Additional file 1 SPIRIT checklist) and Consolidated Standards of Reporting Trials (CONSORT) guidelines [26].

### Participants

Patients will include those with end-stage OA who are on the waiting list at St. Vincent’s Hospital Melbourne, Australia (SVHM) and scheduled to undergo TKA. After patients have seen the orthopaedic surgeon and provided consent for TKA, they attend the orthopaedic preadmission clinic for medical optimisation prior to surgery. Potential patients will be identified and recruited for the study during their attendance at this clinic, where they undergo health assessment and education. The team comprises an orthopaedic surgeon, registrar medical officer and nursing and allied health staff. Eligible patients will include those who have had a previous TKA, in order to determine whether knowledge and experience gained through prior TKA will influence their response to the DCE activity. Orthopaedic surgeons and registrars will include those who perform TKAs at SVHM.

#### Inclusion criteria

These are (1) patients on the surgical waiting list for primary TKA for end-stage OA at SVHM, and (2) orthopaedic surgeons/registrars who consult at the orthopaedic clinics at SVHM.

#### Exclusion criteria

These are (1) patients undergoing revision surgery or surgery for neoplastic disease, and (2) inability to provide informed consent due to mental incompetence (e.g. intellectual disability, dementia).

### Intervention

Patients in the intervention arm will complete a survey containing the DCE. The DCE includes items about postoperative pain, stiffness, quality of life, complications and adverse events (health states) following knee replacement surgery. The remainder of the survey includes items about how much improvement in symptoms patients expect following knee surgery, the level of control, attitude towards taking risks, and the physical and emotional experiences associated with knee pain. Participants will be required to complete the survey prior to TKA during their routine preadmission appointment at SVHM. The survey will take approximately 30 min to complete. Patients will also be required to complete a brief patient expectation survey 1 week prior to scheduled TKA, which will take approximately 5 min to complete. Participants will complete the surveys electronically during their preadmission appointment. Surveys will be completed using a portable computer with administrative assistance provided from the study coordinator.

### Discrete choice experiment development

#### Attributes and levels

To determine attributes and their levels, we used (1) previous literature, (2) data from an existing consecutive cohort of patients who underwent primary elective TKA at SVHM between January 2006 and December 2012 (*N* = 2221, the St Vincent’s Melbourne Arthroplasty Outcomes (SMART) Registry), (3) 40 face-to-face patient interviews, and (4) feedback from experts in the field of orthopaedics and DCE development, including a professor of orthopaedics and Head of the Department of Surgery at SVMH; a professor of economics and Head of the Health Economics Research Programme at the University of Melbourne; the Head of Arthritis Research for the Department of Orthopaedics at SVHM; a rheumatologist at SVHM; and a general practitioner (GP). This resulted in six attributes, with three levels defined for each attribute (Table 1). Two attributes represented pain, two attributes represented physical function, and two attributes represented complications/risks associated with TKA.

**Table 1:** Attributes and levels included in the discrete choice experiment

Attributes	Levels
Pain outcomes:	
1. Day-time pain 9–12 months after surgery	No day-time pain, moderate day-time pain, severe day-time pain
2. Night-time pain 9–12 months after surgery	No night-time pain, moderate night-time pain, severe night-time pain
Functional outcomes:	
3. Standing and walking on a flat surface 9–12 months after surgery	No difficulty standing and walking;
	Moderate difficulty standing and walking, severe difficulty standing and walking
4. Bending to floor, rising from sitting, going up and down stairs 9–12 months after surgery	No difficulty bending to floor, rising from sitting, going up and down stairs
	Moderate difficulty bending to floor, rising from sitting, going up and down stairs
	Severe difficulty bending to floor, rising from sitting, going up and down stairs
Risk of complications:	
5. Risk of having to go back into hospital and have a second operation on your knee	0 %, 7 %, 13 %
6. Risk of getting a complication that requires seeing GP or specialist for further treatment	0 %, 10 %, 21 %

For complication/risk attributes, data were derived from the SMART registry and used to identify the probabilities of the most frequent surgical risks and complications that occurred within 12 months of joint replacement surgery. Specifically, attributes for adverse events were subdivided into two categories: ‘Risk of having to go back into hospital and have a second operation on your knee’ and ‘Risk of getting a complication that requires seeing your GP or specialist for further treatment’. The levels for these absolute risk attributes were based on the minimum, median, and maximum rate of identified risks over the period of the SMART registry from 2006–2012.

For attributes related to measures of treatment efficacy in terms of pain and function following TKA, these were based on items derived from the Western Ontario and McMaster Universities (WOMAC) Osteoarthritis Index [27]. This is a widely used and validated questionnaire specifically designed to evaluate the response to knee OA treatment. Levels for these attributes were derived from existing data in the SMART registry of patient WOMAC scores at 12 months post surgery from all elective primary TKAs performed during 2012 at SVHM (*N* = 331).

#### DCE experimental design

The six attributes and corresponding levels described in Table 1 give rise to a possible combination of 729 (3^6^) treatment outcome scenarios. As this full factorial design of possible scenarios was not feasible to present to each patient, a sample was generated using a fractional factorial experimental design. An efficient design was generated using NGENE software, such that attributes were varied independently from one another across the scenarios and that standard errors were minimised. This model allows for multiple versions of the questionnaire, reducing the burden on patients and increasing the statistical efficiency of the study. Two versions of the DCE questionnaire were generated, each containing six different choice sets. Each DCE questionnaire had the same number of scenarios with the same attributes, but different attribute levels in each questionnaire. The sample of scenarios were then organised into pairwise profiles labelled ‘Choice A’ and ‘Choice B’, and participants were asked to choose between the pair of choices in each of the six choice sets. Furthermore, an opt-out option was included at the end of each choice set where patients were asked if, given the scenarios presented, they would still have the operation or prefer to remain in their current health state. This opt-out option was included as it reflects the voluntary nature of elective TKA in real life. For an example of a discrete choice task see Table 2.

**Table 2:**
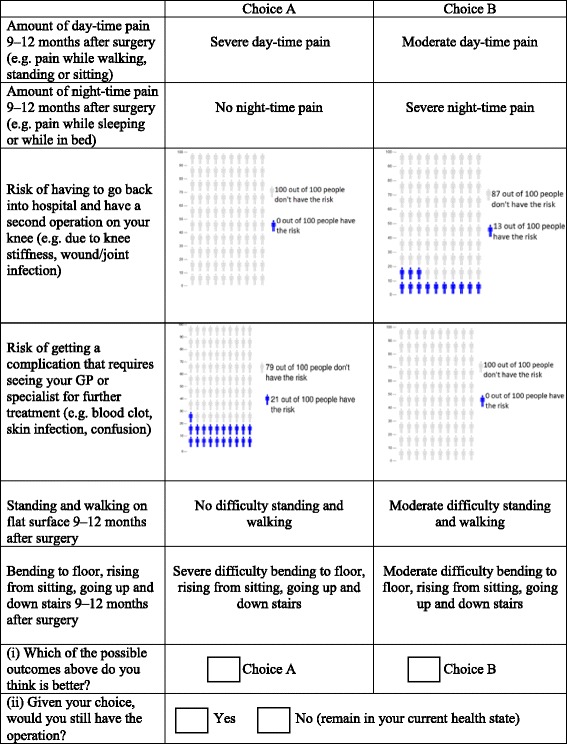
Example of a discrete choice task

#### Pretesting

The DCE survey instrument was pretested to verify the precise wording and framing of attributes and levels. The salience of efficacy figures (i.e. icon arrays) and the wording used to represent rates of adverse events was also tested for ease of comprehension. We also ascertained whether participants could manage the length of the questionnaire. Initial pretesting involved detailed face-to-face interviews of 15 patients. Secondary testing involved the administration of the pilot survey to 40 patients. Participants for pretesting were drawn from patients with end-stage OA who were waitlisted for primary TKA at SVHM. Patients were approached during their attendance at the orthopaedic preoperative assessment clinic at SVHM after consent for TKA by an orthopaedic surgeon. The main issue raised by patients was related to the timing of scenarios. The DCE task was based on possible scenarios postoperatively where patients were asked to imagine that they have had their knee replacement surgery. The detailed interviews revealed that many patients tended to relate the attributes to their current health state. Therefore, the wording of attributes was changed to reinforce the importance of post-surgery in the final design of the survey. Overall, patients felt that the DCE covered all important aspects relating to the postoperative period following knee replacement surgery.

### Standard of care

Patients will undergo surgery and postoperative care as per SVHM’s routine TKA programme which has been standardised through the use of clinical pathway protocols and validated in a RCT [28]. Postdischarge rehabilitation involves either an in-patient or a home-based physiotherapy programme which is predetermined during preadmission clinic assessment using a validated discharge predictor tool [29]. Both programmes are standardised and conducted through SVHM’s in-patient or Hospital-In-The-Home service and are followed by referral to a community-based physiotherapy programme, based on the locality of the patient’s residence. At the preadmission clinic, all patients receive an instruction booklet outlining the continuum of care for TKA. All patients are reviewed post surgery in the same clinic at intervals of 6 weeks, and 3, 6 and 12 months and annually thereafter.

### Outcome assessments

#### Data capture

Demographic information collected at baseline will include age, gender, comorbidities, body mass index and socioeconomic data.

***Primary outcomes***: will be (1) changes in patient-reported knee pain and disability between baseline and 12 months post TKA, and (2) patient satisfaction.

Pain and physical function will be measured on the WOMAC Osteoarthritis Index [27]. The WOMAC consists of 24 items covering three subscales: pain (five items), stiffness (two items) and physical function (17 items). The pain and physical function subscales will be used, each subscale transformed to a score ranging from 0 to 100, with a higher score indicating greater pain and physical function. The WOMAC is a widely used questionnaire specifically designed to evaluate knee and hip OA [30, 31]. Patient satisfaction will be derived from the Self-administered Patient Satisfaction Scale (SAPS) for primary knee arthroplasty, a validated self-administered patient satisfaction scale [30]. Patients will be mailed both questionnaires at 12 months and patients who do not attend review clinics will complete the post-TKA surveys via phone call from a study coordinator, who will be blinded to intervention allocation.

***Secondary outcomes***: will be (1) changes in patient-reported psychological wellbeing between baseline and 12 months post TKA. Psychological wellbeing will be derived from the 12-item short form version of the Veterans RAND Health Survey mental component score (VR-12) [31], (2) differences in expectations of recovery between baseline and 1 week prior to scheduled TKA for patients in the intervention arm. Recovery expectations following TKA will be measured using the 19-item Hospital for Special Surgery: Knee Surgery Expectations Survey [32]. This measure forms a part of the larger study survey, but is measured before patients complete the DCE component, (3) concordance between matched surgeon and patient expectation for recovery following TKA. Surgeons will complete the same expectation survey as patients immediately following consultation and consent for surgery. The surgeon and patient expectation survey will then be paired for analysis.

#### Additional measures

The following independent (predictor) variables will be assessed prior to surgery at baseline during patient preadmission appointments:

- Personality: measured using the Big Five Personality Inventory, a 15-item questionnaire [33]
- Control: measured using the Locus of Control, a 7-item questionnaire [34]
- Risk Attitudes: single-item scale used in the British Household Panel Survey [35]
- Optimism: measured using the 10-item Life Orientation Test-revised Questionnaire [36]
- Fear avoidance beliefs: measured using the 5-item Fear Avoidance Beliefs Questionnaire [37]
- Catastrophising: measured using the 13-item Pain Catastrophising Scale [38]
- Charlson Comorbidity Score (CCS) [39]. This is a weighted index for classifying comorbidities’ severity, validated for estimating the risk of morbidity and mortality in longitudinal studies
- Charnley classification [40]: stratifies patients by the presence of arthritis in one or more large joints; a condition that impairs walking; previous total joint arthroplasty (TJA) on the contra-lateral knee and/or hips
- Baseline radiographic disease severity as assessed by the Kellgren-Lawrence grading [41]

### Timelines

This is a 1-year study with an anticipated start date of February 2016 and end date of January 2017. See Table 3 for SPIRIT schedule of study including enrolment, interventions and assessments.

**Table 3:** SPIRIT trial study schedule

Activity/assessment	Enrolment	Randomisation	*t*_*1*_	*t*_*2*_	*t*_*3*_	*t*_*4*_
	−*t*_*1*_	0				
Baseline data, demographics	X					
Eligibility screen	X					
Informed consent	X					
Randomisation		X				
Baseline assessments battery^a^			X			
Expectation survey^b^				X		
Total knee arthroplasty					X	
12-month assessments^c^						X

^a^Baseline assessments will be given to patients during their preadmission appointments and completed with the assistance of the study coordinator. Assessment items include: Western Ontario and McMaster Universities (WOMAC) Osteoarthritis Index; Veterans RAND Health Survey mental component score (VR-12); Big Five Personality Inventory; Locus of Control; Risk Attitudes; Hospital for Special Surgery: Knee Surgery Expectations Survey; Fear Avoidance Beliefs Questionnaire; Pain Catastrophising Scale; Life Orientation Test-revised Questionnaire; and the DCE survey for patients in the intervention group

^b^Patients will complete the Hospital for Special Surgery: Knee Surgery Expectations Survey 1 week prior to scheduled TKA via phone with study coordinator

^c^12-month assessment will be given to patients at their 1-year post-TKA surgical review visit. Assessments include: WOMAC; VR-12 (this assessment is routine for all patients undergoing TKA)

### Sample size

Intervention effectiveness will be evaluated by comparing change in the primary outcome measure between groups. We aim to detect minimum clinically important improvement (MCII) in the WOMAC. The sample size calculation was based on the following parameters: (1) an alpha value = 0.05, two-sided; (2) power = 80 %. To demonstrate a minimum clinically important difference in WOMAC scores of 15 points (SD [42]), between groups, the sample size required for each group is 45.

To demonstrate a difference in satisfaction the sample size calculation was based on the following parameters: (1) an alpha value = 0.05, two-sided; (2) power = 80 %; (3) expected rates of satisfaction at 1 year post TKA of 77 % for patients in the intervention arm prior to consenting to surgery compared to 51 % for patients undergoing standard procedural consent. The expected rates of satisfaction are derived from a recent study which reported 49 % of patients whose expectations were not met reported dissatisfaction with their TKA compared 6 % of patients whose expectations were met [13]. Incomplete data were reported in 17 % of the patient cohort and, therefore, these patients were not included in the analysis. We have, therefore, assumed a worst case scenario that these 17 % would have reported dissatisfaction despite having their expectations met. Based on these rates the sample size required in each group is 60. To allow for a 10 % dropout of patients, we will recruit 132 patients in total (66 per arm). This estimate is conservative based on our prior RCTs in TKA [43, 44], where we have achieved >95 % retention at 12 months.

### Recruitment

Patients will be approached about participation in the study after consent for TKA, but before surgery. We chose to administer the DCE activity post consent for surgery so as to maintain surgeon blinding and avoid any influence the DCE activity may have on the consent process itself. It is possible but unlikely that completing the DCE activity may influence the decision to undergo TKA. Since we are using an intent-to-treat design, should this occur our statistical analyses will account for missing data by using multiple imputations. Potential patients waitlisted for TKA identified as meeting the inclusion criteria will receive both verbal and written information about the requirements for participation including: (1) verbal explanation of the project by the study coordinator upon initial contact during their attendance at the orthopaedic preadmission clinic at SVHM, (2) a detailed outline of the project content and required commitment in the Patient Information and Consent Form (see Additional file 2: Participant Information and Consent Form). Patients agreeing to take part in the trial will be required to provide written informed consent. Informed consent will be obtained by the research associate only after the patient has read and fully understands the details of the project.

### Randomisation and masking

Following study consent, eligible patients will be randomly assigned in a ratio of 1:1 to either complete the intervention survey or the control survey prior to surgery. Block randomisation will be performed by a computer-generated random assignment sequence prepared in advance. Opaque, numbered, tamperproof envelopes containing assignment will be prepared. Considerable effort will be made to avoid observer bias through separation of roles and blinding of trial staff. A research assistant independent of patient recruitment and data collection will be responsible for patient management. The research associate (who will be responsible for patient consent) will be blinded to group allocation. The surgeons involved in the consent of patients for TKA will have no role in the assignment process. Consenting surgeons will be blinded to patient allocation. In addition, outcome ascertainment will be blinded. Upon completion of the study, a biostatistician blinded to group allocation will analyse outcome data.

### Data management

All data will be stored on a password-protected computer kept in a secure locked facility and only accessible to the chief investigators and the trial coordinator as approved by the SVHM Human Research Ethics Committee (HREC). At the completion of the study, outcome data will be pooled and deidentified for analysis by a statistician. Due to the short duration and minimal risks of the trial, there will not be a data monitoring committee. However, the chief investigator will be responsible for overseeing the trial and ensuring data quality and completeness, including participant enrolment, consent eligibility and forms, allocation to study groups, data recording and timeliness of data collection. Furthermore, there will be no planned interim analyses and stopping guidelines.

### Statistical analysis

Statistical analysis will be by intention-to-treat. Categorical variables will be analysed using chi-squared tests (or Fisher’s exact test for small samples) while continuous variables will be analysed using *t* tests (parametric) and Mann-Whitney (non-parametric) tests for symmetrically and asymmetrically distributed data, respectively. The significance of differences in dichotomous data will be tested using generalised estimating equations or a linear mixed model. If there are chance imbalances in baseline patient characteristics hypothesised to influence the main outcomes, then statistical techniques that allow adjustment for confounding variables will be used as secondary analyses. DCE data will be analysed based on extensions to logistic regression, including multinomial logit models, mixed logit models and generalised multinomial logit models [45]. If there is more than 5 % missing data, sensitivity analysis allowing for different assumptions, such as the best or worst possible scenario, will also be reported for the main outcomes of the study. Cohen’s kappa coefficient will be used to assess concordance between surgeon and patient expectations.

## Discussion

The outcomes of patients following TKA are beneficial on average; however, research demonstrates that patient dissatisfaction following TKA is between 15 and 30 % [13]. One of the strongest predictors of patient dissatisfaction following TKA is unmet patient expectations [21], suggesting a misalignment between patients and surgeons during the procedural consent process. Therefore, it is important to assess patients’ preferences of acceptable risk in undergoing TKA to reduce pain or improve function, which has largely been unexplored. This project aims to use DCEs to explore patient risk-benefit preferences of surgery, and will use a RCT to determine whether applying a DCE prior to surgery improves patient expectations, health outcomes and satisfaction.

DCEs are one of the most commonly used techniques for assessing patients’ preferences in the health care domain. The current research is the first to use a DCE as an intervention to improve patients’ knowledge about the outcomes of a health care intervention. DCEs are not only a useful way to present information, but also require patients to engage through the process of making trade-offs. This may help to ensure that the information presented is more readily absorbed by patients as opposed to simply reading an information leaflet. In recent years there has been an increasing emphasis on the importance of patient involvement in medical decision-making [46–48]. These interventions can improve outcomes by aligning patient and surgeon expectations of surgery, which promotes greater patient satisfaction and increases patient compliance [49].

It is proposed that the DCE validated in the current protocol will aid in better realigning patient expectations with those of their surgeons, which will result in improved knowledge and more realistic expectations for recovery. Better aligning patient and surgeon expectations will, therefore, likely result in improved postoperative health outcomes and satisfaction amongst patients. Reducing the rate of patient dissatisfaction commonly seen in patients following TKA will help to reduce the burden associated with poor outcomes on the health system (i.e. ongoing outpatient consultations, prolonged requirement for allied health services, etc.). Furthermore, exploring whether risk-benefit preferences are influenced by patient characteristics will provide critical information for educating surgeons to understand the anxieties faced by patients and for communicating these to patients undergoing surgery.

## Trial status

Recruitment commencing February 2016.

## Additional files

Additional file 1: SPIRIT checklist. (DOC 121 kb) Additional file 2: Participant Information and Consent Form. (DOCX 26 kb)

## Abbreviations

CONSORT: Consolidated Standards of Reporting Trials
DCE: Discrete choice experiment
OA: Osteoarthritis
OECD: The Organisation for Economic Co-operation and Development
RCT: Randomised controlled trial
SMART: St. Vincent’s Melbourne Arthroplasty Outcomes Registry
SVHM: St. Vincent’s Hospital, Melbourne
TKA: Total knee arthroplasty
