# Supplementary material for: Using discrete choice experiments as a decision aid in total knee arthroplasty: study protocol for a randomised controlled trial
Source: Trials. 2016 Aug 19;17:416. doi: 10.1186/s13063-016-1536-5 (PMC4992237; doi:10.1186/s13063-016-1536-5)
Supplement: Additional file 2: — Participant Information and Consent Form. (DOCX 26 kb) [file 13063_2016_1536_MOESM2_ESM.docx]

**Appendix 1:**

**Participant Information Sheet/Consent Form**

*St. Vincent’s Hospital (Melbourne)*

**Patient Group 2**

| **Title** | *Maximum Acceptable Risk of Complication in Total Knee Arthroplasty (MARKA) Study: using discreet choice experiments to elicit patient and surgeon perception of acceptable risk in total knee arthroplasty* |
| --- | --- |
| **Short Title** | *MARKA Study* |
| **Protocol Number** | *LRR Protocol 175/11* |
| **Project Sponsor** | *NHMRC* |
| **Coordinating Principal Investigator/ Principal Investigator** | *Dr Michelle Dowsey* |
| **Associate Investigator(s)** | *Prof Peter Choong, Prof Anthony Scott,*  *A/Prof Vijaya Sundararajan, Dr Mandana Nikpour*  *Dr Jinhu Li* |
| **Study Co-ordinator**  **Location** | *Dr Elizabeth Nelson*  *St Vincent’s Hospital, Melbourne* |

**Part 1 What does my participation involve?**

**1 Introduction**

You are invited to take part in this research project. This is because you have arthritis of your knee joint and have consented to undergo a total knee replacement at St. Vincent’s Hospital. This research project aims to measure if completing a risk-benefit preference based survey prior to knee replacement surgery will improve patient expectations, health outcomes and satisfaction following surgery compared to undergoing standard procedural consent alone.

This Participant Information Sheet/Consent Form tells you about the research project. It explains the tests and treatments involved. Knowing what is involved will help you decide if you want to take part in the research.

Please read this information carefully. Ask questions about anything that you don’t understand or want to know more about. Before deciding whether or not to take part, you might want to talk about it with a relative, friend or your local doctor.

Participation in this research is voluntary. If you don’t wish to take part, you don’t have to. You will receive the best possible care whether or not you take part.

If you decide you want to take part in the research project, you will be asked to sign the consent section. By signing it you are telling us that you:

• Understand what you have read

• Consent to take part in the research project

• Consent to have the tests and treatments that are described

• Consent to the use of your personal and health information as described.

You will be given a copy of this Participant Information and Consent Form to keep.

**2 What is the purpose of this research?**

Arthritis is one of the leading causes of disability worldwide. The treatment for severe arthritis of the knee is total knee replacement (TKR). Around 45,000 patients undergo TKR in Australia each year. Despite a high overall success rate with this operation, up to 1 in 3 patients who receive a knee replacement still express some dissatisfaction with the level of improvement in their pain and/or function following surgery. Research indicates that the strongest predictor of patient dissatisfaction following TKA is unmet expectations. This is known to occur when patients and surgeons have different expectations of surgery, and it is reported that more than half of patients undergoing TKR report higher expectations than their surgeons.

Since unmet expectations occur commonly amongst patients undergoing TKR, it is important to develop a simple, non-invasive, cost-effective way of facilitating informed choice and decision-making. The completion of a risk-benefit preference based survey prior to knee replacement surgery can act as a simple decision making aid, used to help improve patient knowledge and realistic expectations of upcoming surgery. This may lead to greater satisfaction and appropriateness of care for patients undergoing TKR.

You are invited to participate in this research project because you are scheduled to have routine surgery to replace your knee. This research study will be conducted at St Vincent’s Hospital, Melbourne.

The purpose of this study is to determine if completing a discrete choice experiment (DCE) risk-benefit preference survey prior to surgery will help improve health related outcomes 1-year after TKR. It is expected that a total of 132 patients undergoing total knee replacement will be recruited to this study.

This research has been funded by a National Health and Medical Research Council Project Grant.

**3 What does participation in this research involve?**

Participation in this research will involve the normal procedures preparing you for your knee replacement surgery. This includes your attendance at a clinic before surgery (pre-admission clinic), where you will be approached about participation in the study by the study coordinator.

You will be participating in a randomised controlled trial, which is regarded as the best way of evaluating if an intervention is effective. In these types of studies, patients are put into different groups and each group is given a different intervention. To try to make sure the groups are the same at the beginning of the study, each participant is allocated to a group by chance (randomised).

In this study, there will be two different groups, a “test” group and a “control” group. A roughly even number of participants will be randomised into each group. All patients participating in this study will be provided with the usual care for patients having TKR at St. Vincent’s Hospital Melbourne; the only difference will be the completion or not of the risk-benefit preference based survey prior to surgery. One group, the test group, will complete a survey containing the risk-benefit preference decision aiding tool. The control group will complete a modified survey that does not contain the risk-benefit preference activity. Participants in both groups will be required to complete a survey prior to TKR during their routine pre-admission appointment at SVHM. Patients will also be required to complete a brief patient expectation survey 1-week prior to scheduled TKR. All trial patients will be followed up at the one year post-op period. However, it is routine for all patients undergoing knee replacement at St Vincent’s Hospital to be reviewed at one year post-surgery, where a measure of health related outcomes is completed.

The test group survey will include items about post-operative pain, stiffness, quality of life, complications and adverse events (health states) following the TKR. These items are organised into a number of scenarios and then organised into pairs, where you will be asked to choose between a number of these pairs of choices. Both test group and control group surveys include items about how much improvement in symptoms you expect following knee surgery, the level of control you have over things in your life, and your attitude towards taking risks, and the physical and emotional experiences associated with knee pain. Surveys can be completed electronically or paper-based hardcopy. Electronic surveys will be completed using a portable computer with administrative assistance provided from the study coordinator. We expect this survey to take approximately 30 minutes.

Information about your medical history and demographics including age, gender, medical conditions, socioeconomic data and cultural linguistic background, and x-rays will also be collected from your medical records.

There are no additional costs associated with participating in this research project, nor will you be paid.

**4 What do I have to do?**

The research team from St Vincent’s Hospital will provide you with all the information you require to participate in this study. You should continue to take any medications prescribed to you by your General Practitioner, or other doctors, and you will be able to receive any other medical treatments you require during the study period.

**5 Other relevant information about the research project**

This study will be conducted at St Vincent’s Hospital Melbourne. One hundred and thirty two participants will be involved in the study over a one year period, with approximately 66 patients in the test group and control group.

The study is collaboration between the Department of Orthopaedic Surgery at St Vincent’s Hospital and the Melbourne Institute of Applied Economic and Social Research at the University of Melbourne.

**6 Do I have to take part in this research project?**

Participation in any research project is voluntary. If you do not wish to take part, you do not have to. If you decide to take part and later change your mind, you are free to withdraw from the project at any stage.

If you do decide to take part, you will be given this Participant Information and Consent Form to sign and you will be given a copy to keep.

Your decision whether to take part or not to take part, or to take part and then withdraw, will not affect your routine treatment, your relationship with those treating you or your relationship with St Vincent’s Hospital.

**7 What are the possible benefits of taking part?**

There will be no obvious benefit to you as an individual from participation in this research. We hope that results from this study will improve planning for others who require knee replacement surgery. Your participation in this project will help lead to improved decision making tools for improving patient knowledge and realistic expectations of surgery.

**8 What are the possible risks and disadvantages of taking part?**

The project will require your time and commitment, with possible discomfort involving the time to complete the survey. It is not expected that there will be harm caused to you by being involved in this project. If you become upset or distressed as a result of your participation in the research, the researchers are able to arrange for counselling or other appropriate support. Any counselling or support will be provided by qualified staff who are not members of the research project team. This counselling will be provided free of charge. You may end your participation in the research at any time if distress occurs.

**9 What if new information arises during this research project?**

During the research project, new information about the risks and benefits of the project may become known to the researchers. If this occurs, you will be told about this new information and the researcher will discuss whether this new information affects you.

**10 What if I withdraw from this research project?**

If you decide to withdraw from the project, please notify a member of the research team before you withdraw. Your ongoing care at St Vincent’s Hospital Melbourne will not be jeopardised in any way should you choose to withdraw from this study

If you do decide to leave the project, the researchers would like to keep the personal and health information already collected about you. This is to help them make sure that the results of the research can be measured properly. If you do not want them to do this, you should tell them at the time you withdraw from the project.

**11 Could this research project be stopped unexpectedly?**

We do not anticipate that this research project will be stopped unexpectedly. In this very unlikely situation you would be advised immediately and your ongoing care would continue in a standard fashion at St Vincent’s Hospital Melbourne.

**12 What happens when the research project ends?**

At the completion of this research project you will continue to receive the standard of care at St Vincent’s Hospital Melbourne.

**Part 2 How is the research project being conducted?**

**13 What will happen to information about me?**

All data obtained during this research study will be stored at the Department of Orthopaedics, St Vincent’s Hospital, Melbourne. Only the research team will have access to data collected as a part of this research. Any information obtained in connection with this project, including that from your medical records, will remain confidential. Any identifiable data will be stored securely - either locked in the department or password protected if kept as a computer copy. It will only be disclosed with your permission. You are entitled to access all data regarding your own information upon request. The results will be held for 15 years after which time they will be destroyed. The results of the research may be published in a scientific journal in such, but any such publication would not contain identifiable data, thereby maintaining your privacy and confidentiality.

If you give us your permission by signing the Consent Form, we plan to discuss and share the results with the scientific community to enhance further knowledge and understanding in total joint replacement surgery. By signing the attached consent form you authorize release of, and access to, this confidential information to the relevant study personnel.

Information about your participation in this research project will be recorded in your health records.

In accordance with relevant Australian and/or Victorian privacy and other relevant laws, you have the right to request access to your information collected and stored by the research team. You also have the right to request that any information with which you disagree be corrected. Please contact the study team member named at the end of this document if you would like to access your information.

**14 Complaints and compensation**

If you suffer any injuries or complications as a result of this research project, you will continue to be treated by the Orthopaedic team at St Vincent’s Hospital Melbourne.

If you have any complaints regarding your treatment while participating in this trial you will be able to speak to a representative of St Vincent’s Hospital Melbourne, the Patient Liaison Officer, who has no role in the current research trial nor the Department of Orthopaedics.

**15 Who is organising and funding the research?**

This research project is being conducted by St Vincent’s Hospital, Melbourne and is being funded by the National Health and Medical Research Council.

No member of the research team will receive a personal financial benefit from your involvement in this research project (other than their ordinary wages).

**16 Who has reviewed the research project?**

All research in Australia involving humans is reviewed by an independent group of people called a Human Research Ethics Committee (HREC). The ethical aspects of this research project have been approved by the HREC of St Vincent’s Hospital, Melbourne.

This project will be carried out according to the *National Statement on Ethical Conduct in Human Research (2007)*. This statement has been developed to protect the interests of people who agree to participate in human research studies.

**17 For further information and who to contact**

**For further information or appointment**

The person you may need to contact will depend on the nature of your query.

If you want any further information concerning this project or appointments you can contact the study co-ordinator: Dr Elizabeth Nelson, Study Co-ordinator, St. Vincent’s Hospital (Melbourne) on Telephone: (03) 9231 3516.

***Complaints:***

If you have any complaints about any aspect of the study or the way in which it is being conducted you may contact the Patient Liaison Officer at St Vincent’s Hospital (Melbourne) on Telephone: (03) 9231 3108. You will need to tell the Patient Liaison Officer the name of the person who is noted above as principal investigator.

***Research Participant Rights:***

If you have any questions about your rights as a research participant, then you may contact the Executive Officer Research at St Vincent’s Hospital (Melbourne) on Telephone: (03) 9231 3930.

**Consent Form -** *Adult providing own consent*

| **Title** | *Maximum Acceptable Risk of Complication in Total Knee Arthroplasty (MARKA) Study: using discreet choice experiments to elicit patient and surgeon perception of acceptable risk in total knee arthroplasty* |
| --- | --- |
| **Short Title** | *MARKA Study* |
| **Protocol Number** | *LRR Protocol* |
| **Project Sponsor** | *NHMRC* |
| **Coordinating Principal Investigator/**  **Principal Investigator** | *Dr Michelle Dowsey* |
| **Associate Investigator(s)** | *Prof Peter Choong, Prof Anthony Scott,*  *A/Prof Vijaya Sundararajan, Dr Mandana Nikpour*  *Dr Jinhu Li* |
| **Location** | *St Vincent’s Hospital, Melbourne* |

**Declaration by Participant**

I have read the Participant Information Sheet or someone has read it to me in a language that I understand.

I understand the purposes, procedures and risks of the research described in the project.

I give permission for my doctors, other health professionals, hospitals or laboratories outside this hospital to release information to St Vincent’s Hospital concerning my disease and treatment for the purposes of this project. I understand that such information will remain confidential.

I have had an opportunity to ask questions and I am satisfied with the answers I have received.

I freely agree to participate in this research project as described and understand that I am free to withdraw at any time during the study without affecting my future health care.

I understand that I will be given a signed copy of this document to keep.

|  | | | | | | | | |
| --- | --- | --- | --- | --- | --- | --- | --- | --- |
|  | Name of Participant (please print) | |  |  |  | | |  |
|  | | | | | | | | |
|  | Signature |  | | Date | | ___________ |  |  |

**Declaration by Study Researcher**

I have given a verbal explanation of the research project, its procedures and risks and I believe that the participant has understood that explanation.

|  | | | | | | |
| --- | --- | --- | --- | --- | --- | --- |
|  | Name of Researcher (please print) | |  | | |  |
|  | | | | | |  |
|  | Signature |  | | Date |  |  |
